# A complete map of potential pathogenicity markers of avian influenza virus subtype H5 predicted from 11 expressed proteins

**DOI:** 10.1186/s12866-015-0465-x

**Published:** 2015-06-26

**Authors:** Zeeshan Khaliq, Mikael Leijon, Sándor Belák, Jan Komorowski

**Affiliations:** Department of Cell and Molecular Biology, Computational and Systems Biology, Science for Life Laboratory, Uppsala University, SE-751 24 Uppsala, Sweden; Department of Virology, Parasitology and Immunobiology (VIP), National Veterinary Institute (SVA), Uppsala, Sweden; OIE Collaborating Centre for the Biotechnology-based Diagnosis of Infectious Diseases in Veterinary Medicine, Ulls väg 2B and 26, SE-756 89 Uppsala, Sweden; Department of Biomedical Sciences and Veterinary Public Health (BVF), Swedish University of Agricultural Sciences (SLU), Uppsala, Sweden; Institute of Computer Science, Polish Academy of Sciences, 01-248 Warszawa, Poland

**Keywords:** Avian Influenza virus, Pathogenicity, Virulence, MCFS, Rosetta, Rough sets

## Abstract

**Background:**

Polybasic cleavage sites of the hemagglutinin (HA) proteins are considered to be the most important determinants indicating virulence of the avian influenza viruses (AIV). However, evidence is accumulating that these sites alone are not sufficient to establish high pathogenicity. There need to exist other sites located on the HA protein outside the cleavage site or on the other proteins expressed by AIV that contribute to the pathogenicity.

**Results:**

We employed rule-based computational modeling to construct a map, with high statistical significance, of amino acid (AA) residues associated to pathogenicity in 11 proteins of the H5 type viruses. We found potential markers of pathogenicity in all of the 11 proteins expressed by the H5 type of AIV. AA mutations S-43^HA1^-D, D-83^HA1^-A in HA; S-269-D, E-41-H in NA; S-48-N, K-212-N in NS1; V-166-A in M1; G-14-E in M2; K-77-R, S-377-N in NP; and Q-48-P in PB1-F2 were identified as having a potential to shift the pathogenicity from low to high. Our results suggest that the low pathogenicity is common to most of the subtypes of the H5 AIV while the high pathogenicity is specific to each subtype. The models were developed using public data and validated on new, unseen sequences.

**Conclusions:**

Our models explicitly define a viral genetic background required for the virus to be highly pathogenic and thus confirm the hypothesis of the presence of pathogenicity markers beyond the cleavage site.

**Electronic supplementary material:**

The online version of this article (doi:10.1186/s12866-015-0465-x) contains supplementary material, which is available to authorized users.

## Background

Highly pathogenic avian influenza viruses (HPAIV) pose a threat for yet another epidemic or pandemic, which can potentially result in severe consequences for both animal and human life. So far, only the low pathogenic avian influenza viruses (LPAIV) of H5 and H7 serotypes have been shown to be precursors for the HPAIV’s [1, 2]. To infect, the surface glycoprotein hemagglutinin (HA) precursor, HA0, needs to be cleaved by cellular proteases into functional HA1 and HA2 subunits [3]. LPAIV’s carry a monobasic cleavage site that is recognized only by trypsin-like proteases [4, 3, 5] thus limiting the infection to the respiratory and gastrointestinal tracts [3, 5]. High pathogenicity has been previously linked to insertions in the cleavage site of HA [6–8]. These insertions allow the HA0 to be cleaved by ubiquitously expressed intracellular proteases such as furin [9–11] leading to a systematic infection and lethal disease with mortality rates being as high as 100 %. Recent studies [12–14] have shown that the insertions in the cleavage site of HA may not be sufficient to render the virus highly pathogenic (HP). Although the cleavage site certainly is the most important virulence determinant, evidence is accumulating that the HPAIV’s also carry virulence determinants other than the polybasic cleavage sites in the HA protein [3, 5, 4]. Such determinants may be located outside the cleavage site of the HA protein and spread across all the expressed proteins of the virus creating a suitable environment for high pathogenicity [3]. Since only H5 and H7 subtypes are known to become HP and since there are too few HP sequences of the H7 subtypes for our analysis, we limited our study to the analysis of H5 serotype of the AIV.

**Fig. 1:**
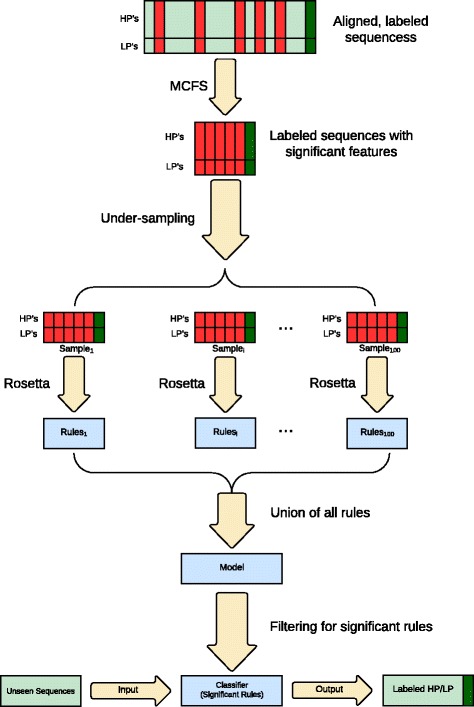
Schematic representation of the applied computational modeling methodology

We used publicly available protein sequence data for all the proteins of H5 subtype of avian influenza viruses (H5-AIV) [15]. Pathogenicity is very strongly linked to the amino acid sequence of the cleavage site for naturally occurring viruses [16] and thus aligned sequences of the proteins were annotated with the pathogenicity value (high or low) using the presence or absence of insertions in the cleavage site of the corresponding HA protein, respectively. The cleavage site was subsequently removed from the HA protein sequences since we had already used this information to label the sequences. This enables learning other AA positions of the sequences that may be related to the pathogenicity label. Ranking of the pathogenicity significant AA positions for each of the proteins was done with Monte Carlo Feature Selection (MCFS) [17]. Rough set theory [18] as implemented by ROSETTA [19] was applied in constructing rule-based models of pathogenicity using the significant positions. Such models are expressed as IF-THEN rules. See Fig. 1 for a schematic description of the method. The rules explicitly specified AA’s and their combinations that were associated with the pathogenicity of the H5 subtype. In addition to already known markers of pathogenicity, we discovered other potential AA positions and mutations that may affect the pathogenicity of H5-AIV. The models were experimentally validated on new, unseen sequences released after we built our models. Similar approaches to modeling that we used here have been successfully applied to model many aspects of protein or gene features such as, for instance, cleavability of octamer peptides by HIV-1 protease [20], drug resistance [21], binding affinities [22] and participation in biological processes [23].

To the best of our knowledge this work is the first proteome-wide characterization of the pathogenicity landscape of the H5 subtype of avian influenza viruses based on all available sequences to date.

## Results

### High quality predictive models of pathogenicity for all proteins

Following MCFS that identified AA positions significant in discriminating high from low pathogenic (LP) sequences (Table 1, Additional file 1: Table S1 and Additional file 2: MCFS_output.xlsx), we constructed high quality predictive models of pathogenicity of the H5 viruses expressed in the form of IF-THEN rules. Application of MCFS was essential in processing this high dimensional data and provided ranking of the importance of the AA positions with respect to discriminating pathogenicity. ROSETTA was applied to construct rule-based models for each of the proteins using the significant features as selected by MCFS as described in Material and Methods.

**Table 1:** The training data

Protein	H5N1		Non-H5N1		Total		All Features	Significant Features
	HP	LP	HP	LP	HP	LP		
HA	1377	54	48	512	1425	566	616	82
NA	551	32	23	264	574	296	593	114
M1	161	9	13	52	174	61	329	16
M2	186	9	14	63	200	72	98	18
NS1	425	16	22	148	447	164	249	71
NS2	202	3	14	53	216	56	129	25
NP	294	12	22	113	316	125	511	22
PA	465	22	25	235	490	257	730	57
PB1	405	26	25	223	430	249	775	44
PB2	446	26	23	247	469	273	783	62
PB1-F2	135	16	15	114	150	130	101	40

The HP and LP columns represent the number of highly pathogenic and low pathogenic sequences in each of the proteins, respectively. The ‘All features’ column is the total number of features (*i.e.* AA’s) from which significant features are selected with Monte Carlo Feature Selection

**Fig. 2:**
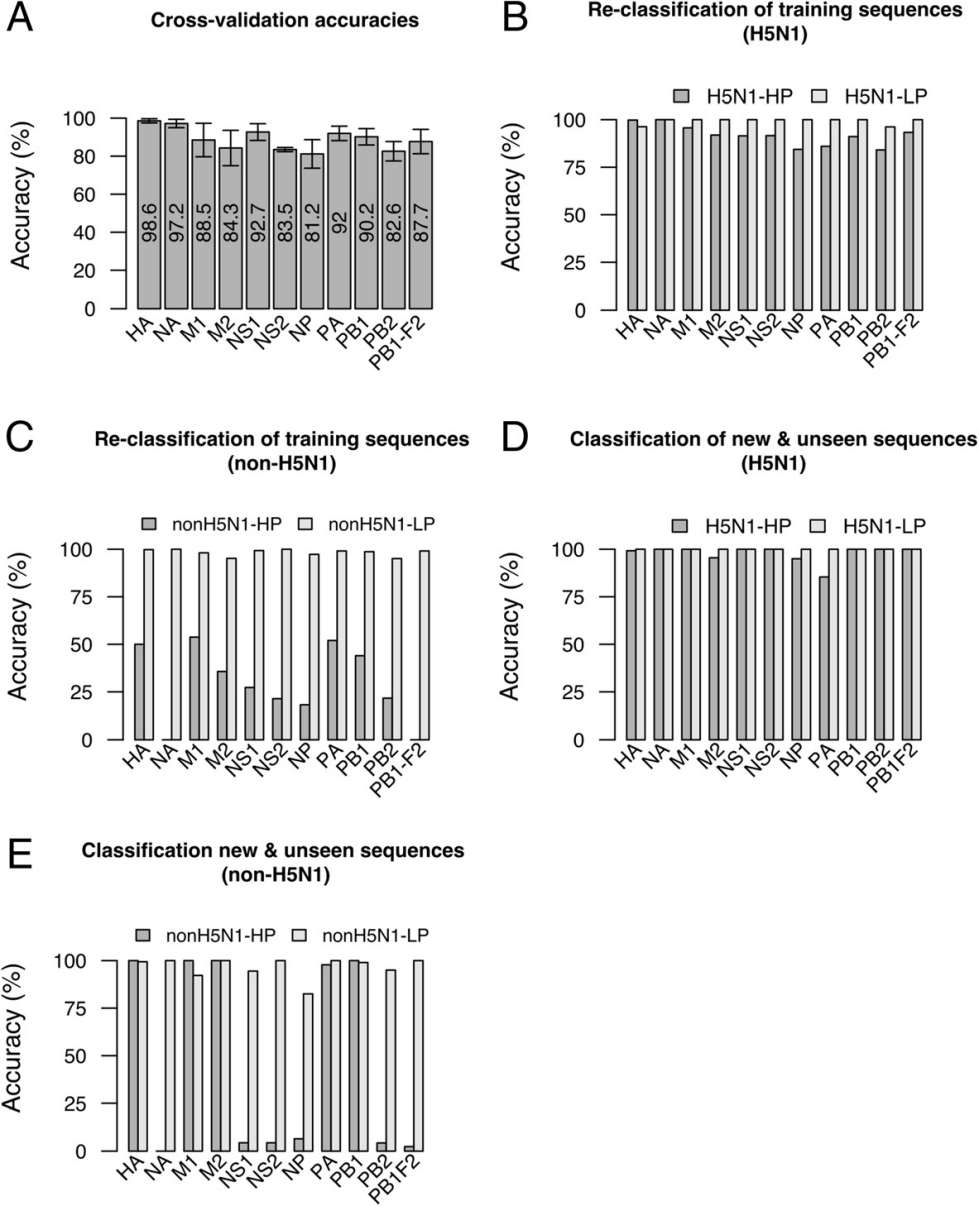
Accuracies of the cross-validation and the testing of the models on new, unseen data. **a** Quality measures for the rule-based models. Averaged Accuracy is the average of mean accuracy from the 10-fold cross-validation loop for the models created on 100 under-sampled subsets for each protein. Standard deviation from the 10-fold cross validation loop, averaged in a similar way as accuracy, is shown as error bars on the plot. **b** Re-classification of the training sequences of the H5N1 sequences. Accuracy is the percentage of correctly classified sequences. See also Additional file 4: Table S2. **c** Re-classification of the training sequences of the non-H5N1 sequences. Accuracy is the percentage of correctly classified sequences. See also Additional file 4: Table S3. **d** Accuracies of the classifiers when tested on the newly published unseen H5N1 sequences, *i.e.* sequences not included in the training of the models and with sequences identical to the training sequences removed. Accuracy is the percentage of correctly classified sequences. Classifiers consisted of the significant rules from all the rule-based models created for a given protein. See also Additional file 5: Table S4. **e** Accuracies of the classifiers when tested on the newly published unseen non-H5N1 sequences, *i.e.* sequences not included in the training of the models and with sequences identical to the training sequences removed. Accuracy is the percentage of correctly classified sequences. Classifiers consisted of the significant rules from all the rule-based models created for a given protein. See also Additional file 5: Table S5

The model for the HA protein, with the cleavage site removed, was the best one with 98.6 % accuracy and 1.1 % standard deviation, while the models for the other proteins had accuracies in the range 81.2–97.2 %, with the least accurate model for the NP protein (Fig. 2a).

#### Extraction of significant rules from the models

For the HA protein the set of significant rules (*p* < 0.05; hyper-geometric distribution; Bonferroni-corrected p-value) consisted of 138 rules for high pathogenicity and 65 rules for low pathogenicity. Similarly, for the NA protein we obtained 11 rules for high pathogenicity and 59 for low pathogenicity. The sets of significant rules are referred to as classifiers in our study. The classifiers for all the proteins are listed in Additional file 3: Classifiers.xlsx.

### High pathogenicity rules in the classifiers were highly accurate for the H5N1 subtype sequences

Since the HP training sequences were predominantly of the H5N1 subtype (Table 1) (*e.g.* HA had 1377 H5N1 sequences out of 1425), we suspected that the rules for high pathogenicity would predominantly be learning H5N1 pathogenicity. This hypothesis was confirmed through re-classification of the training sequences by the classifiers. For the HA protein, 1374 of the 1377 H5N1 HP sequences were correctly re-classified as HP, and 52 of the 54 H5N1 LP sequences were correctly re-classified as LP. From the total of 48 HP sequences of the non-H5N1 subtypes, we could only correctly re-classify 24 sequences. However, 511 of the 512 non-H5N1 LP sequences were re-classified correctly (Fig. 2b and c, Additional file 4: Table S2-S3).

For the NA protein, all of the 551 H5N1 HP sequences were classified correctly as HP and all 32 of the 32 H5N1 LP sequences were also classified correctly. None of the 23 non-H5N1 HP sequences were correctly classified, while all of the 264 non-H5N1 LP sequences were correctly classified. The numbers for the remainder of the proteins are shown in Fig. 2b and c (see also Additional file 4: Table S2-S3).

### Validation of classifiers on new, unseen sequences

Our classifiers were generated using data published on 23 January 2014. We validated our classifiers by classifying new, unseen sequences published after that date. Only unique sequences were considered, *i.e.*, sequences identical to the sequences used in learning the models were removed. The cleavage site in these new sequences was removed prior to classification.

For the H5N1 subtype sequences, the HA rules performed with accuracy of 99.3 % classifying 134 of 135 sequences correctly (Fig. 2d, Additional file 5: Table S4). Rules from the NA models classified all of the 108 sequences correctly *i.e.* accuracy 100 %. Rules from M1, NS1, NS2, PB1, PB1-F2 and PB2 gave a perfect classification of the sequences *i.e.* accuracy was 100 %. M2 rules classified 47 of 49 sequences correctly and NP rules classified 42 of 44 sequences correctly. Rules from the PA models were the least accurate with 85.4 % accuracy by correctly classifying 39 of 45 sequences.

The non-H5N1 type HP sequences were mostly of the H5N8 subtype (HA: 43 of 48, NA: 43 of 44, M1: 42 of 43, M2: 41 of 42, NS1: 43 of 45, NS2: 43 of 45, NP: 43 of 46, PA: 43 of 45, PB1: 43 of 45, PB2: 43 of 46, PB1-F2: 40 of 41) (Fig. 2e, Additional file 5: Table S5). Interestingly, the classifiers for HA, M1, M2 and PB1 proteins gave a perfect classification (100 % accuracy) for the respective protein sequences. 45 of 46 new PA sequences were also correctly classified. On the other hand only two of the 45 for NS1 and NS2 sequences each, three of the 46 NP sequences, two of 46 PB2 sequences and 1 of 41 PB1-F2 sequences could be classified correctly with our classifiers. It suggests that the pathogenicity markers carried by these new H5N8 sequences in the HA, M1, M2, PB1 and PA protein are similar to the ones for the H5N1 subtypes.

### Amino acids and their combinations associated with pathogenicity

Filtering for the strongest rules (*Accuracy* ≥ 80 % and *Class-Specific-Coverage* ≥ 50 %) in the HA classifier produced three rules for high pathogenicity and 13 for low pathogenicity (Table 2). The HP HA rules associated D-43^HA1^, A-83^HA1^ and I-71^HA1^ with high pathogenicity. The LP-rules associated S-43^HA1^, D-83^HA1^, S-107^HA1^, N-138^HA1^, D-309^HA1^, V-302^HA1^, A-7^SP^, I-6^SP^, D-275^HA1^, N-195^HA1^, S-240^HA1^, R-3^SP^ and S-194^HA1^ with low pathogenicity, where HA1 and HA2 are the two subunits of HA and SP is the signal peptide of the non-cleaved sequence. For the NA protein, from the strongest rules (Additional file 6: Table S6), AA residues N-369, G-386, T-288, D-269, H-41 and H-100 were associated with high pathogenicity and N-400, K-38, V-192, P-90, I-73, I-262, L-255, M-24, S-14, E-41, S-269, K-187, T-434, E-74 and S-43 were associated with low pathogenicity. The strongest rules for the other proteins are shown in Additional file 6: Table S7-S16. AA’s and their combinations associated with high and low pathogenicity, respectively, for all the proteins are summarized in Table 3.

**Table 2:** The strongest rules for highly and low pathogenic viruses from the HA classifier

	Rule	Accuracy (%)	Support	Class-Specific-Coverage (%)
HP-Rules	IF P43(HA1) = D THEN virus = HP	99.8	1225	86
	IF P83(HA1) = A THEN virus = HP	100.0	807	57
	IF P71(HA1) = I THEN virus = HP	100.0	759	53
LP-Rules	IF P43(HA1) = S THEN virus = LP	95.2	589	99
	IF P83(HA1) = D THEN virus = LP	94.6	571	95
	IF P107(HA1) = S THEN virus = LP	95.8	552	93
	IF P138(HA1) = N THEN virus = LP	92.7	536	88
	IF P309(HA1) = D THEN virus = LP	94.9	533	89
	IF P320(HA1) = V THEN virus = LP	95.7	532	90
	IF P195(HA1) = N THEN virus = LP	88.8	400	63
	IF P16(SP) = G THEN virus = LP	89.3	392	62
	IF P203(HA2) = I THEN virus = LP	82.4	380	55
	IF P6(SP) = I THEN virus = LP	97.5	354	61
	IF P7(SP) = A THEN virus = LP	98.0	352	61
	IF P3(SP) = R THEN virus = LP	94.1	341	57
	IF P240(HA1) = S THEN virus = LP	95.2	332	56
	IF P275(HA1) = D THEN virus = LP	97.3	300	52

Accuracy is the percentage of the sequences in the support set correctly classified by the rule. Support is the number of sequences that satisfy the “IF” conditions of the rule. Class-Specific-Coverage is the percentage per class (*i.e.* HP or LP, respectively) of the sequences that support the rule and are correctly classified by the rule. For instance, if a rule is an HP class rule then the Class-Specific-Coverage gives the percentage of the HP sequences classified correctly by this rule

**Table 3:** AA’s and its combinations associated with high and low pathogenicity in all the proteins

	Association to high pathogenicity		Association to low pathogenicity	
	Singular residues	Combination of residues	Singular residues	Combination of residues
HA	D-43^HA1^, A-83^HA1^, I-71^HA1^	-	S-43^HA1^, D-83^HA1^, S-107^HA1^, N-138^HA1^, D-309^HA1^, V-302^HA1^, A-7^sp^, I-6^sp^, D-275^HA1^, N-195^HA1^, S-240^HA1^, R-3^SP^, S-194^HA1^	-
NA	N-369, G-386, T-288, H-100, D-269, H-41	-	N-400, K-38, V-192, P-90, I-73, I-262, L-255, M-24, S-14, E-41, S-269, K-187, T-434, E-74, S-43	-
NS1	N-48, L-207, N-212	R-59 & N-212,	M-27	P-208 & K-212,
		F-22 & N-48		S-82 & R-113 & D-166,
				F-22 & S-82 & T-89 & R-113,
				P55 = E & P73 = S & P82 = S & P89 = T & P113 = R
				S-48 & S-73 & S-82 & D-166,
				S-48 & S-82 & D-166,
				S-82 & A-107 & R-113,
				S-73 & S-82 & R-113,
				S-82 & R-113,
				S-82 & K-212
NS2	A-22, A-115, V-14	V-6 & I-60	-	V-49 & S-60
M1	A-166, N-232, N-224, K-27, I-168	T-121 & I-168	V-166, R-101	V-166 & D-232
M2	E-14	-	-	G-14 & E-66,
				G-14 & I-28,
				G-14 & K-18,
				I-28 & S-82,
				K-18 & I-28
NP	S-34	N-377 & N-482,	N-450	K-77 & V-353 & S-377
		S-34 & N-377,		
		S-34 & N-482,		
		R-77 & N-482,		
		R-77 & N-377,		
		A-373 & S-450,		
		A-373 & N-377		
PA	T-129, S-58	-	-	-
PB1	I-149, V-14, L-384	I-113 & I-149,	-	A-14 & V-113 & G-154 & S-3
		V-14 & I-113,		
		I-113 & K-386,		
		T-59 & 113-I & K-215,		
		I-113 & K215		
PB2	M-64, T-339	-	I-478	M-64 & I-478
PB1-F2	Y-57, P-48	-	Q-48, D-50	-

AA’s for the HA protein are shown to be either of HA1, HA2 or Signal Peptide (SP). AA’s for the NA protein shown are numbered according to the whole length sequences *i.e.* the sequences without the stalk deletion

### AA mutations associated with the shift of pathogenicity from low to high

To associate AA mutations with a shift in pathogenicity from low to high we compared the strongest HP and LP rules that have the same position but a different residue. There were two such cases in the strongest HA rules.

For the HA protein, all the LP rules that specified position 43^HA1^ had a Serine residue and the HP rules with position 43^HA1^ had an Aspartic acid residue there. Hence, the mutation S-43^HA1^-D was associated with a shift in pathogenicity from low to high. Similarly, mutation D-83^HA1^-A was also associated with a pathogenicity shift from low to high. By the same token, mutations S-269-D, E-41-H in the NA protein, S-48-N, K-212-N in the NS1 protein, V-166-A in the M1 protein, G-14-E in the M2 protein, K-77-R, S-377-N in the NP protein and Q-48-P in the PB1-F2 protein (Table 4) were associated with shifts of pathogenicity from low to high.

**Table 4:** AA mutations associated with a shift of pathogenicity from low to high

	AA mutations associated with a change in pathogenicity from low to high
HA	S-43^HA1^-D, D-83^HA1^-A
NA	S-269-D, E-41-H
NS1	S-48-N, K-212-N
NS2	-
M1	V-166-A
M2	G-14-E
NP	K-77-R, S-377-N
PA	-
PB1	-
PB2	-
PB1-F2	Q-48-P

### Analysis of the AA alterations involved in the strongest rules

An analysis of AA positions in the rules associated to pathogenicity (Table 3) produced the following results.

### HA rules

**Fig. 3:**
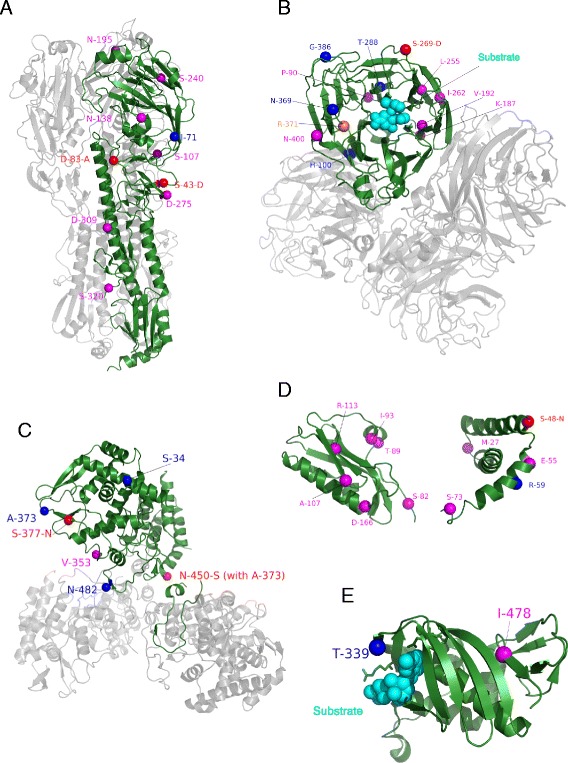
AA’s appearing in the most significant rules marked on the 3D structures of different proteins. AA residues appearing in the rules are shown as spheres. Positions from the high pathogenicity rules are shown in blue, positions from the low pathogenicity rules are in magenta and mutations associated with the shift of pathogenicity from low to high as defined by the rules are shown in red. **a** Mapping of amino acid positions associated with pathogenicity from the rules onto 3D structure of the HA protein of Influenza A virus (A/Hubei/1/2010 (H5N1)) (PDB: 4KTH). Chain A (HA1 residues) and chain B (HA2 residues) are presented in green, while the rest of the trimer is shown in gray. **b** A cartoon representation of chains A, B, C and D of the NA protein with AA positions from the rules (PDBID: 2HU4). Chain A, the one marked with rule positions, is shown in green and the others in gray. Residue R-371, shown as a sphere in orange, is a part of the catalytic site of the protein. Cyan spheres constitute Oseltamivir 2, a substrate bound to the protein. **c** A cartoon representation of the NP protein trimer (PDBID: 2IQH) with positions from the rules. Chain A is shown in green and the others are in gray. **d** AA’s from the rules marked on a cartoon representation of NS1 (PDBID: 3FST). **e** A cartoon representation of the PB2 protein cap-binding domain (PDBID: 4CB4) with AA’s from the rules

AA residues at positions 138 and 212 appearing in the LP rules and AA residue at position 108, adjacent to position 107 from our rules, have previously been linked with pathogenicity [24]. AA residue at position 320^HA1^, appearing in the LP rules, is a residue flanking the cleavage site on one side and is also shown previously to affect pathogenicity [25]. AA residues at positions 42^HA1^ and 274^HA1^, which are adjacent to residues at positions 43^HA1^ and 275^HA1^ appearing in our rules, make a di-sulfide bond (UniProt: O56140). AA positions from the strongest rules for HA are shown in Fig. 3a.

### NA rules

Position 369 appearing in the HP rules lays close to position 371 of the active site of the protein as shown in Fig. 3b. Positions 369, 288, 386, 269, 400, 434 and 187 are on the surface of the protein (Fig. 3b). Position 100 is in the region that is in contact with chain B of the NA tetramer. AA residue at position 400 from the LP rules is close to a potential glycosylation site at position 402 [26].

### NP rules

Positions 377, 482, 373 and 450 appears in the PB2 interaction domain (AA 340–498) of NP [27]. More specifically, position 482 is found in the last 33 amino acids of NP that regulate the NP-PB2 binding [27]. Positions 34 and 77 are in a domain that is characterized as RNA binding sub-region (AA 1–77) [28] and PB2 interaction domain (AA 1–161) [27]. Position 34 occurring in the HP rules has previously been shown to be a determinant of temperature sensitivity [29]. All the positions in the rules were on the surface of the protein (Fig. 3c).

### NS1 rules

AA residue at position 48 from the HP rules lays next to position 49, which has been shown to be involved in RNA binding [30]. AA residue at position 212 occurs in the binding site of the adapter protein Crk/CrkL-SH3 domain [31]. Residues at positions 207 from the HP rules and 208 from the LP rules occur in an unstructured and flexible tail region which contains a number of motifs including Crk/CrkL-SH3 binding and PDZ ligand [32]. Residues at position 82 and 113 from the LP rules are positioned at the ends of the eIF4G1-binding domain [33], which stretches from residue 81–113. Position 89 of the NS1 protein appearing in the LP rules has been shown to be one of the residues on NS1 protein where p85β, a regulatory subunit of phosphatidylinositol-3-kinase (PI3K), binds [34]. AA residue at position 107 is adjacent to 106, which has been shown to affect the replication rate in mammalian cells [35]. Positions from the strongest rules are shown in Fig. 3d.

### NS2 rules

Position 22, appearing in the strongest HP rules for the NS2 protein, is positioned at the end of the nuclear export signal motif while position 14 is inside it, which stretches from 12 to 21 (UniProt: O56263). Position 49 in the strongest HP rules is near position 47, which has been previously reported to be related to pathogenicity [36].

### M1 rules

Positions 166, 232, 224 and 168 appearing in the HP rules are in the C-terminal domain that binds to viral RNP [37] while position 121 is in a lipid binding site [37].

### M2 rules

Position 14 appearing in M2 rules is adjacent to a conserved W-15 residue in a highly mutable region (aa10-20) of the M2 extracellular domain [38]. Position 18, in the LP rules, is situated in the M2 extracellular domain. The AA residue at position 28 is adjacent to position 27, which has been shown in H3 and H1 subtypes to be associated with adamantane resistance [39, 40].

### PA rules

Positions 58 and 129 appearing in our rules are part of the N-terminus region of PA, which is considered to be the most active part of the protein. Position 58 is next to position 57, which has been shown to be involved in suppressing the host protein synthesis during infection [41].

### PB1 rules

Position 14 is in the PA binding domain (AA 1–25) of the PB1 protein [42]. It is next to position 13, which is considered to be a determinant of the host range for AIV’s [43]. Position 215 is situated in the nuclear localization motif (AA 203–216) and positions 384 and 386 are found in the catalytic domain of the protein (UniProt-Q9Q0V0).

### PB2 rules

Position 64, next to position 63, from our rules has been shown to be a determinant of pathogenicity [44]. Position 339 is in the cap-binding domain AA 318–483 (Fig. 3e) [45]. Interestingly, our rules use position 627. Previously, mutation E-627-K has been shown to increase pathogenicity [46]. However, it occurred in only 18 % of the HP sequences of this study and consequently was not selected to be sufficiently significant by our Class-Specific-Coverage filtering that was set for all the rules to be at least 50 % (see Experimental Procedures).

### PB1-F2 rules

Position 57 is next to position 56 that has been shown to affect pathogenicity in H5N1 mallard ducks [47].

## Discussion

The goal of this study was to identify AA’s and their combinations other than the cleavage site of the HA protein that have the potential to affect the pathogenicity of the H5 subtype of AIV. Another goal was to associate specific AA changes or mutations with a shift of pathogenicity from low to high. To this end, mathematical modeling using known methods of Monte Carlo Feature Selection and rough sets were applied to all the viral proteins. Sets of statistically significant rules were extracted from the models and called classifiers. Validation of the classifiers performed on new, unseen sequences, *i.e.* sequences published after we extracted the data for our analysis, showed that the model was indeed very highly predictive for at least the period of 10 months, *i.e.* between January and October 2014.

Among the identified positions, the most interesting seemed to be the ones that defined mutations associated with the shift from LP to HP in several of the proteins. The positions and their residue values defined the genetic background of the AIV necessary to produce a high pathogenicity as defined by the characteristics of the HA cleavage site. This finding was consistent with the idea of pathogenicity markers outside the cleavage site of the HA protein [13, 3, 14, 5, 12, 4].

The results of re-classification showed that the rules of the classifiers for high pathogenicity were able to correctly and with very high accuracy classify H5N1 HP sequences, while non-H5N1 HP sequences were classified poorly. However, regardless of the subtype, the re-classification of all LP sequences was done with high accuracy although the prevalence of H5N1 LP was low, as opposed to the prevalence of non-H5N1 LP sequences. This suggests that the mechanism of low pathogenicity is similar in all H5 type sequences, while the mechanism of high pathogenicity of H5N1 is specific to this subtype.

Furthermore, it is interesting to notice that the new non-H5N1 HP sequences were mostly of the H5N8 subtype that caused outbreaks in South Korea earlier this year [48]. Following genetic analysis, this reassortant virus is thought to have borrowed HA, PB2 and NP segments from H5N1-like viruses, PB1, PA, M and NS segment from H4N2-like viruses, and NA from the previous H5N8-like avian influenza viruses [48]. We observed a perfect classification by the models of HA, M1, M2, PB1 and PA but poor classification by the rest of the models. Notably, the PB2 and NP protein models performed poorly even though these segments are thought to be contributed by H5N1-like viruses with which we trained the models. This may suggest that the PB2 and NP segments have adapted to H5N8-specific highly pathogenic viral background. The perfect classification of the M1, M2, PB1 and PA models, despite them being contributed by H4N2-like viruses, suggests that the markers of high pathogenicity in these proteins are similar to the H5N1 type markers. It would follow that the viral background necessary for high pathogenicity in the new H5N8 viruses is partly (*i.e.* in HA, PA, PB1, M1 and M2 proteins) similar to that of the H5N1-like viruses. Nevertheless, the fact that this background differs in the NS1, NS2, PB2, PB1-F2, NP and NA proteins supports our conclusion that the markers of high pathogenicity are subtype specific.

A survey of the AA’s, which have been shown to be important for the H5 pathogenicity in previous studies, suggests that our methods pinpoint biologically relevant locations in various viral proteins. Our positions were, among others, in the immediate neighborhood of positions that have been indicated previously, in the vicinity of active sites of the proteins and in a few cases were a direct hit of a position that have been described earlier as important for pathogenicity. However, since most of the published results have not been done in the context of pathogenicity the comparisons needs to be treated with caution. To the best of our knowledge, our findings are the first ones to be based on a large number of available sequence data and are statistically significant.

However, it eventually will be necessary to confirm these findings experimentally not only by testing them on new, unseen sequences, but also by testing specific combinations of mutations to find out minimal sets that induce high pathogenicity. Our computational work clearly shows where to search and makes this search plausible by a dramatic restriction to a manageable number of cases. Further studies of the particular roles of these novel markers outside the polybasic cleavage site of the HA protein, and on the other proteins, as well as the roles of activating proteases in various hosts, affecting these newly detected marker sites, will be required.

## Conclusions

We used sequences of all 11 proteins of the avian influenza A virus to build high quality and easy to read IF-THEN rule-based models of pathogenicity for each protein. From the rules we extracted a map of markers of both high and low pathogenicity in all the proteins. Our models were able to correctly predict low pathogenicity independently of the NA subtype indicating that low pathogenicity was common to all H5 viruses irrespectively of NA type. The same was not true for high pathogenicity where we could only correctly classify the HP H5N1 sequences with high accuracies (100 % for some proteins) but not the HP non-H5N1 sequences. Surprisingly, the pathogenicity of the new and unseen H5N8 subtype sequences, that are currently circulating, could be perfectly predicted with the H5N1 models of HA, M1, M2, PA and PB1 and poorly with the models for the other proteins. This suggests that the pathogenicity markers of the H5N8-like viruses are similar to H5N1-like viruses in these proteins but different from H5N1-like viruses in the rest of the proteins. In summary, we identified a viral background for the H5N1 type viruses that, in addition to the previously known insertions in the cleavage site of the hemagglutinin protein, seems to be necessary for a virus to become highly pathogenic. This study narrows down the possible combinatorial space for pathogenicity analysis and provides a platform for further analysis and biological verification of the discovered pathogenicity markers. The detection and identification of the additional sites and factors of virulence alteration in the viral genomes provide basic novel information for the i) better understanding of viral evolution; ii) for the improvement of diagnosis; iii) and for the development of more effective vaccine candidates and other measures to control the devastating diseases caused by AIV all over the world.

## Methods

### Amino acid sequences and their alignment

The data was downloaded from the NCBI Influenza Virus Resource database [15] in January 2014 as FASTA files. It contained unique AA sequences from the 11 AIV proteins: HA, NA, M1, M2, NS1, NS2, NP, PA, PB1, PB2 and PB1-F2. To download the HA sequences, we filled the form at the NCBI’s influenza resource download page as follows:

- Type: A
- Host: Avian
- Country/Region: any
- Protein: HA
- Subtype: H: any N: any
- Full length plus: checked
- Collapse identical sequences: checked

The “collapse identical sequences” option guaranteed we did not have identical sequences in our data sets. All the other sequences for the other proteins were also downloaded with the same settings. At this point the data contained all subtypes. The downloaded amino acid sequences were aligned using MUSCLE (v3.8.31) [49], for each protein separately. These aligned sequences were used to create decision tables. The H5-type sequences were then extracted from the decision tables as described in the next section.

### Annotation and extraction of data of interest

For each protein, the data was organized into a decision table: the first column contained the identifiers of the AA sequences, the following columns represented positions and contained the corresponding AA’s with the last column to contain the outcome, *i.e.* the pathogenicity label 1 or 0, where 1 labeled a highly pathogenic (HP) sequence and 0 a low pathogenic (LP) one, respectively. Gaps in the alignment were represented by a ‘?’ in the decision tables. The rows of a decision table are called objects, while columns other than the first and the last one are called features.

Pathogenicity is very strongly linked to the amino acid sequence of the cleavage site for naturally occurring viruses [16]. For a sequence to be labeled as HP, two criteria must be met; 1) There is an elongation of the cleavage site, *i.e.* an insertion of one or more AA’s in the cleavage site; 2) The last 4 AA’s of the cleavage site must be of the form (R/K)XX(R/K) [16].

Since the pathogenicity values were not present in the NCBI database, we labeled each sequence according to the criteria as defined above. Sequences with no corresponding HA sequence information could not be labeled and were not included in the analysis. Importantly, the sequence corresponding to the cleavage sites in the HA protein was removed prior to further analysis. From the decision tables, we extracted AA sequences belonging to the H5 serotype.

### Feature selection

To remove noise from the data and to select only the features that contributed significantly towards discerning high from low pathogenicity, *Monte Carlo Feature Selection* (MCFS) was used as described in [17], which is implemented in dmLab [50]. MCFS uses a very large number of decision trees to assess the contribution of features (AA positions) towards the outcome (HP/LP). MCFS computes a normalized relative importance (RI-norm) score for each feature. Statistical significance of the RI-norm scores was assessed with a permutation test and significant features (p < 0.05), after Bonferroni correction [51], were kept as described in [52]. Only these significant features were used in the further mathematical model generation.

### Rough sets and rule-based model generation

Rough set theory [18] was used as the basis for mathematical modeling. Rough sets produced minimal sets of features that can discern between the objects belonging to different decision classes. The final representation of the discernibility is in the form of IF-THEN rules. ROSETTA [19], a publicly available software system [53] (http://www.lcb.uu.se/tools/rosetta/) that implements rough sets theory, was used to build rule-based models. A complete description of rough sets can be found in [54] and the combined MCFS-ROSETTA approach to model generation in bioinformatics is described in [55].

The input data to ROSETTA were decision tables containing the viral sequences using only the significant features that were from feature selection. ROSETTA computed approximately minimal subsets of feature combinations that discern between the outcomes HP and LP. The classifiers were collections of IF-THEN rules that were inferred from the labeled AA sequences. A sample rule

**Table:** 

Rule	*Accuracy (%)*	*Support*
IF P22 = F AND P48 = N THEN virus = HP	99.7	355

reads: “**IF***at position 22 there is a Phenylalanine residue***AND***at position 48 there is an Asparagine residue***THEN***the virus is highly pathogenic*”.

There is additional information about the rules available, too. For this rule, *Accuracy* is 99.7 %, that is the proportion of true positives and true negatives to the sum of true positives, true negatives, false positives and false negatives is 0.997. *Support* is the set of sequences (355 sequences) that satisfy the conditions of the left hand side (LHS), *i.e.* the set of sequences that have a Phenylalanine residue at position 22 and an Asparagine residue at position 48. The above rule is a conjunctive rule since there is a conjunction of conditions (P22 = F AND P48 = N) in the left hand side (LHS) of the rule. A rule can also be a singleton rule where LHS consists of only a single condition.

In the data for all the proteins, the number of HP and LP sequences differed significantly in favor of the HP sequences. This imbalance would affect the learning process in favor of the class having more objects [56]. One of the solutions to this problem was to balance the classes [57]. To address this problem a technique called under-sampling was used. The set of all HP objects was sampled randomly to create 100 subsets, where the number of HP sequences was equal to the number of LP sequences effectively producing balanced data sets. A single rule-based model was inferred from each of the subsets, which resulted in 100 models per protein. We illustrate the process with the following example.

The data set of the HA protein had 1425 HP and 566 LP sequences, which was a significant imbalance in the number of sequences. From the HP set we created subsets by randomly extracting 100 times 566 HP objects and joining them with the 566 LP objects to create 100 subsets. A rule-based model was inferred from each of the subsets. To assess the performance of the models, a 10-fold cross validation was performed for each of them. Mean accuracy and accuracy standard deviation were calculated for all the models. For each protein the average of the accuracies and standard deviations for the 100 models served as the quality assessment criteria for our rule-based models.

### Filtering the most significant rules (classifiers) from all the models for a protein

Significant rules (p < 0.05; hyper-geometric distribution; Bonferroni-corrected p-value, as described previously) were filtered from the 100 models. These rules were called classifiers.

### Validation of the classifiers

In order to validate our models on new, unseen data, we downloaded H5N1 type AA sequences for each protein from the NCBI website [15] that were made available after we constructed our models. These sequences were classified with our classifiers.

### Extraction of the strongest rules from our classifiers

To extract the strongest rules from each classifier, for each of the significant rule in the classifiers we calculated *Class-Specific-Coverage*, which was the percentage of correctly classified viral sequences in the HP and LP class, respectively, according to the formula:

$$ Class- Specific- Coverage=\frac{\left( Accuracy\times Support\right)}{TotalSequences}\times 100 $$

*Support* is the number sequences that satisfy the LHS conditions of the rule, as described earlier, and *Accuracy* is the accuracy of the rule.

If the rule for which we intended to calculate *Class-Specific-Coverage* was for the HP class then *TotalSequences* was the total number of the HP sequences and if the rule was for the LP class then it was the total number of the LP sequences. After calculating *Class-Specific-Coverage* for all the rules in a classifier for each protein, we extracted rules that had *Accuracy* of at least 80 % and *Class-Specific-Coverage* of at least 50 %.

### From alignment positions to true positions

The AA positions occurring in rules of our classifiers for all the proteins were corresponding to the positions coming from their respective multiple alignments. In order to compare the AA’s occurring in our rules to the ones that have already been discussed in literature, we needed to obtain the true positions. A true position in a single sequence would be the position without counting the alignment gaps. For each AA position occurring in the rules in our classifiers, for all proteins, we identified its corresponding true position. This was achieved as follows. We took an AA position appearing in rules from a classifier and identified its corresponding true position in each of the training sequence used to infer the classifier. The position to which our alignment position mapped in most of sequences was taken to be the true position. For example, to obtain the corresponding true position for position 169 (alignment position) in the rules for the M1 protein, we found that it corresponded to position 166 in 226 sequences and position 165 in nine sequences. Hence we said alignment position 169 of the M1 protein corresponds to true position 166.

Positions 1–16 of HA were labeled as signal peptide (SP) positions. Positions 17–336 were labeled as HA1 subunit positions and the remaining positions were labeled as positions of the HA2 subunit. For the HA protein we showed true positions for each subunit, respectively. For example, true position 2^HA1^ corresponded to true position 18 of the HA and true positions 2^HA2^ corresponded to true position 338 after the cleavage site is removed from the sequence.

For the NA protein we showed rules with AA positions for the full-length protein (without deletion in its stalk region). In the Additional file 6: Table S16 we also provided rules with positions for the NA protein that has a deletion in its stalk region.

### Classification of an AA sequence

For a given AA sequence, all the rules with conditions matching the sequence are called to fire for the sequence. Each rule has a decision class in the THEN-part. The firing rule is called to vote for the decision. The number of votes is equal to the support set of the rule. All the votes of all the firing rules are summed up per class and the majority wins. As a sample application consider a sequence MALAMTRS and the following (hypothetical) classifier with 5 rules:

1. IF P3 = L THEN virus = HP, Support = 16
2. IF P4 = A AND P7 = R THEN virus = HP, Support = 10
3. IF P6 = K AND P7 = R THEN virus = HP, Support = 7
4. IF P3 = S THEN virus = LP, Support = 12
5. IF P5 = M AND P8 = S THEN virus = LP, Support = 6

Rules 1, 2 and 5 fire for the sequence casting 16, 10 and 6 votes, respectively, resulting in 26 votes for class HP and 6 votes for class LP. Rules 3 and 4 do not fire and are not considered any further. The majority of votes is for HP and the classifier thus determines the sequence to belong to this class. Clearly, the AND in the condition of a rule means that all the conjuncts must be satisfied in order for the rule to fire. It is also important to note that a single rule by itself cannot be used to infer pathogenicity. Classification is obtained only by an application of all rules of the classifier.

Furthermore, if the conditions of a rule are not met for a sequence, it cannot be inferred that the sequence belongs to the other class or classes than the one given by the THEN-part. It further follows that rules provide only positive evidence. If there is no rule in the classifier that would fire for a given sequence, its virulence status remains unknown.

### Scripting programming language

Python programming language was used for scripting purposes.

## Additional files

Additional file 1: Table S1. A table showing the top 20 features selection by the feature selection for HA protein. Additional file 2:
**Spreadsheet that contains the MCFS output for all the proteins.**
Additional file 3:
**Spreadsheet containing complete classifiers for all the proteins.**
Additional file 4:
**Contains** **Table S2** **and Table S3** **that show the performance of re-classification of the training data.**
Additional file 5:
**Contains Table S4-S5 showing the performance of our models of new, unseen H5N1 and non-H5N1 type sequences.**
Additional file 6:
**Contains Table S6-S16 that shows the strongest rules from the classifiers of the proteins other than HA.**

## Abbreviations

AA: Amino acids
AIV: Avian influenza viruses
HA: Hemagglutinin
HPAIV: Highly pathogenic avian influenza viruses
HP: Highly pathogenic
H5-AIV: H5 subtype of avian influenza viruses
LP: Low pathogenic
LPAIV: Low pathogenic avian influenza viruses
M1: Matrix protein 1
M2: Matrix protein 2
MCFS: Monte carlo feature selection
NA: Neuraminidase
NP: Nucleoprotein
NS1: Non structural protein 1
NS2: Non structural protein 2
PA: Polymerase acidic protein
PB1: Polymerase basic protein 1
PB2: Polymerase basic protein 2
