# Supplementary material for: A complete map of potential pathogenicity markers of avian influenza virus subtype H5 predicted from 11 expressed proteins
Source: BMC Microbiol. 2015 Jun 26;15:128. doi: 10.1186/s12866-015-0465-x (PMC4482282; doi:10.1186/s12866-015-0465-x)
Supplement: Additional file 1: Table S1. — A table showing the top 20 features selection by the feature selection for HA protein. [file 12866_2015_465_MOESM1_ESM.docx]

Table S1: Top 20 significant features for the HA protein after Monte Carlo Feature Selection sorted by the RI-norm score. See also Additional file 3: MCFS_output.xlsx.

*RI-norm* is the normalized relative information score, which is calculated for each attribute in MCFS. This RI-norm can be seen as the discriminatory power of a feature. For details see Draminski, M., Rada-Iglesias, A., Enroth, S., Wadelius, C., Koronacki, J., and Komorowski, J. (2008). Monte Carlo feature selection for supervised classification. Bioinformatics *24*, 110-117.

| **Rank** | **Feature** | **RI-norm**^[[1]](#footnote-1)^ | **P-value** | **Rank** | **Feature** | **RI-norm** | **P-value** |
| --- | --- | --- | --- | --- | --- | --- | --- |
| 1 | P108 (HA1) | 0.8429505 | < 2.7 e-297 | 11 | P88 (HA1) | 0.53548276 | < 2.7 e-297 |
| 2 | P208 (HA2) | 0.82893187 | < 2.7 e-297 | 12 | P320 (HA1) | 0.51469004 | < 2.7 e-297 |
| 3 | P209 (HA1) | 0.784367 | < 2.7 e-297 | 13 | P83 (HA1) | 0.45353293 | < 2.7 e-297 |
| 4 | P68 (HA2) | 0.66225386 | < 2.7 e-297 | 14 | P309 (HA1) | 0.44450954 | < 2.7 e-297 |
| 5 | P43 (HA1) | 0.595391 | < 2.7 e-297 | 15 | P126 (HA1) | 0.40675804 | < 2.7 e-297 |
| 6 | P48 (HA2) | 0.5940935 | < 2.7 e-297 | 16 | P138 (HA1) | 0.33928892 | < 2.7 e-297 |
| 7 | P217 (HA1) | 0.570853 | < 2.7 e-297 | 17 | P212 (HA1) | 0.3256207 | < 2.7 e-297 |
| 8 | P119 (HA1) | 0.56511354 | < 2.7 e-297 | 18 | P45 (HA1) | 0.2942917 | < 2.7 e-297 |
| 9 | P198 (HA1) | 0.55247587 | < 2.7 e-297 | 19 | P169 (HA2) | 0.2804803 | < 2.7 e-297 |
| 10 | P107 (HA1) | 0.5397411 | < 2.7 e-297 | 20 | P124 (HA1) | 0.2229564 | < 2.7 e-297 |

1. [↑](#footnote-ref-1)
