# Supplementary material for: A complete map of potential pathogenicity markers of avian influenza virus subtype H5 predicted from 11 expressed proteins
Source: BMC Microbiol. 2015 Jun 26;15:128. doi: 10.1186/s12866-015-0465-x (PMC4482282; doi:10.1186/s12866-015-0465-x)
Supplement: Additional file 4: — Contains Table S2 and Table S3 that show the performance of re-classification of the training data. [file 12866_2015_465_MOESM4_ESM.docx]

Table S2: Re-classification of the training data H5N1 subtype. Related to Figure 2B.

| **Protein** | **HP** | **Correct classification** | **(%)** | **LP** | **Correct classification** | **(%)** | **Total** | **Correct classification** | **(%)** |
| --- | --- | --- | --- | --- | --- | --- | --- | --- | --- |
| HA | 1377 | 1374 | 99.8 | 54 | 52 | 96.3 | 1431 | 1426 | 99.7 |
| NA | 551 | 551 | 100 | 32 | 32 | 100 | 583 | 583 | 100 |
| M1 | 161 | 154 | 95.7 | 9 | 9 | 100 | 170 | 163 | 95.9 |
| M2 | 186 | 171 | 91.9 | 9 | 9 | 100 | 195 | 180 | 92.3 |
| NS1 | 425 | 389 | 91.5 | 16 | 16 | 100 | 441 | 405 | 91.8 |
| NS2 | 202 | 185 | 91.6 | 3 | 3 | 100 | 205 | 188 | 91.7 |
| NP | 294 | 248 | 84.4 | 12 | 12 | 100 | 306 | 260 | 85 |
| PA | 465 | 400 | 86 | 22 | 22 | 100 | 487 | 422 | 86.7 |
| PB1 | 405 | 369 | 91.1 | 26 | 26 | 100 | 431 | 395 | 91.6 |
| PB2 | 446 | 375 | 84.1 | 26 | 25 | 96.2 | 472 | 400 | 84.7 |
| PB1F2 | 135 | 126 | 93.3 | 16 | 16 | 100 | 151 | 142 | 94 |

Table S3: Re-classification of the training data non-H5N1 subtype. Related to Figure 2C.

| **Protein** | **HP** | **Correct classification** | **(%)** | **LP** | **Correct classification** | **(%)** | **Total** | **Correct classification** | **(%)** |
| --- | --- | --- | --- | --- | --- | --- | --- | --- | --- |
| HA | 48 | 24 | 50 | 512 | 511 | 99.8 | 560 | 535 | 95.5 |
| NA | 23 | 0 | 0 | 264 | 264 | 100 | 287 | 264 | 92 |
| M1 | 13 | 7 | 53.8 | 52 | 51 | 98.1 | 65 | 58 | 89.2 |
| M2 | 14 | 5 | 35.7 | 63 | 60 | 95.2 | 77 | 65 | 84.4 |
| NS1 | 22 | 6 | 27.3 | 148 | 147 | 99.3 | 170 | 153 | 90 |
| NS2 | 14 | 3 | 21.4 | 53 | 53 | 100 | 67 | 56 | 83.6 |
| NP | 22 | 4 | 18.2 | 113 | 110 | 97.3 | 135 | 114 | 84.4 |
| PA | 25 | 13 | 52 | 235 | 233 | 99.1 | 260 | 246 | 94.6 |
| PB1 | 25 | 11 | 44 | 223 | 220 | 98.7 | 248 | 231 | 93.1 |
| PB2 | 23 | 5 | 21.7 | 247 | 235 | 95.1 | 270 | 240 | 88.9 |
| PB1F2 | 15 | 0 | 0 | 114 | 113 | 99.1 | 129 | 113 | 87.6 |
