# Supplementary material for: A complete map of potential pathogenicity markers of avian influenza virus subtype H5 predicted from 11 expressed proteins
Source: BMC Microbiol. 2015 Jun 26;15:128. doi: 10.1186/s12866-015-0465-x (PMC4482282; doi:10.1186/s12866-015-0465-x)
Supplement: Additional file 5: — Contains Table S4-S5 showing the performance of our models of new, unseen H5N1 and non-H5N1 type sequences. [file 12866_2015_465_MOESM5_ESM.docx]

Table S4: Classification of the new, unseen H5N1 type sequences. Related to Figure 2D.

| **Protein** | **HP** | **Correct classification** | **(%)** | **LP** | **Correct classification** | **(%)** | **Total** | **Correct classification** | **(%)** |
| --- | --- | --- | --- | --- | --- | --- | --- | --- | --- |
| HA | 130 | 129 | 99.2 | 5 | 5 | 100 | 135 | 134 | 99.3 |
| NA | 104 | 104 | 100 | 4 | 4 | 100 | 108 | 108 | 100 |
| M1 | 50 | 50 | 100 | 5 | 5 | 100 | 55 | 55 | 100 |
| M2 | 44 | 42 | 95.5 | 5 | 5 | 100 | 49 | 47 | 95.9 |
| NS1 | 45 | 45 | 100 | 5 | 5 | 100 | 50 | 50 | 100 |
| NS2 | 42 | 42 | 100 | 2 | 2 | 100 | 44 | 44 | 100 |
| NP | 40 | 38 | 95 | 4 | 4 | 100 | 44 | 42 | 95.5 |
| PA | 41 | 35 | 85.4 | 4 | 4 | 100 | 45 | 39 | 86.7 |
| PB1 | 47 | 47 | 100 | 4 | 4 | 100 | 51 | 51 | 100 |
| PB2 | 48 | 48 | 100 | 5 | 5 | 100 | 53 | 53 | 100 |
| PB1F2 | 44 | 44 | 100 | 5 | 5 | 100 | 49 | 49 | 100 |

Table S5: Classification of the new, unseen non-H5N1 type sequences. Related to Figure 2E.

| **Protein** | **HP** | **Correct classification** | **(%)** | **LP** | **Correct classification** | **(%)** | **Total** | **Correct classification** | **(%)** |
| --- | --- | --- | --- | --- | --- | --- | --- | --- | --- |
| HA | 48 | 48 | 100 | 162 | 161 | 99.4 | 210 | 209 | 99.5 |
| NA | 44 | 0 | 0 | 101 | 101 | 100 | 145 | 101 | 69.7 |
| M1 | 43 | 43 | 100 | 103 | 95 | 92.2 | 146 | 138 | 94.5 |
| M2 | 42 | 42 | 100 | 90 | 90 | 100 | 132 | 132 | 100 |
| NS1 | 45 | 2 | 4.4 | 91 | 86 | 94.5 | 136 | 88 | 64.7 |
| NS2 | 45 | 2 | 4.4 | 95 | 95 | 100 | 140 | 97 | 69.3 |
| NP | 46 | 3 | 6.5 | 97 | 80 | 82.5 | 143 | 83 | 58 |
| PA | 45 | 44 | 97.8 | 100 | 100 | 100 | 145 | 144 | 99.3 |
| PB1 | 45 | 45 | 100 | 97 | 96 | 99 | 142 | 141 | 99.3 |
| PB2 | 46 | 2 | 4.3 | 101 | 96 | 95 | 147 | 98 | 66.7 |
| PB1F2 | 41 | 1 | 2.4 | 87 | 87 | 100 | 128 | 88 | 68.8 |
