# Supplementary material for: A complete map of potential pathogenicity markers of avian influenza virus subtype H5 predicted from 11 expressed proteins
Source: BMC Microbiol. 2015 Jun 26;15:128. doi: 10.1186/s12866-015-0465-x (PMC4482282; doi:10.1186/s12866-015-0465-x)
Supplement: Additional file 6: — Contains Table S6-S16 that shows the strongest rules from the classifiers of the proteins other than HA. [file 12866_2015_465_MOESM6_ESM.docx]

## Strongest Rules from the classifiers

Tables S6-S16 show the strongest rules from the classifiers for NA, NS1, NS2, M1, M2, NP, PB1, PA, PB2 and PB1-F2 proteins.

Table S6: The strongest rules from the NA classifier.

|  | Rule | Accuracy  (%) | Support | Class-Specific-Coverage (%) |
| --- | --- | --- | --- | --- |
| **HP-Rules** | IF P369=N THEN virus=HP | 100 | 513 | 89 |
|  | IF P386=G THEN virus=HP | 100 | 418 | 73 |
|  | IF P288=T THEN virus=HP | 97.1 | 452 | 76 |
|  | IF P269=D THEN virus=HP | 100 | 364 | 63 |
|  | IF P41=H THEN virus=HP | 100 | 330 | 57 |
|  | IF P100=H THEN virus=HP | 100 | 296 | 52 |
| **LP-Rules** | IF P400=N THEN virus=LP | 93.5 | 277 | 87 |
|  | IF P38=K THEN virus=LP | 94.3 | 264 | 84 |
|  | IF P192=V THEN virus=LP | 95.7 | 253 | 82 |
|  | IF P90=P THEN virus=LP | 92.2 | 270 | 84 |
|  | IF P73=I THEN virus=LP | 93.5 | 245 | 77 |
|  | IF P262=I THEN virus=LP | 93.4 | 242 | 76 |
|  | IF P255=L THEN virus=LP | 93 | 230 | 72 |
|  | IF P24=M THEN virus=LP | 92.5 | 227 | 71 |
|  | IF P14=S THEN virus=LP | 90.3 | 237 | 72 |
|  | IF P41=E THEN virus=LP | 93 | 215 | 68 |
|  | IF P269=S THEN virus=LP | 91 | 223 | 69 |
|  | IF P187=K THEN virus=LP | 91.5 | 211 | 65 |
|  | IF P434=T THEN virus=LP | 94.1 | 188 | 60 |
|  | IF P74=E THEN virus=LP | 92.3 | 194 | 60 |
|  | IF 43=S THEN virus=LP | 91.3 | 195 | 60 |

Table S7: The strongest rules from the NS1 classifier.

Position 22 appears in both HP and LP rules (rule 1 in HP and rule 3 in LP) with the same amino acid. This may be due to the fact that ROSETTA calculates approximately minimal subsets, not absolutely minimal subsets of discerning features.

|  | Rule | Accuracy  (%) | Support | Class-Specific-Coverage (%) |
| --- | --- | --- | --- | --- |
| **HP-Rules** | IF P22=F AND P48=N THEN virus=HP | 99.7 | 355 | 79 |
|  | IF P48=N THEN virus=HP | 98.4 | 374 | 82 |
|  | IF P207=L THEN virus=HP | 99.7 | 347 | 77 |
|  | IF P59=R AND P212=N THEN virus=HP | 100 | 271 | 61 |
|  | IF P212=N THEN virus=HP | 97.7 | 301 | 66 |
| **LP-Rules** | IF P208=P AND P212=K THEN virus=LP | 84.5 | 148 | 76 |
|  | IF P82=S AND P113=R AND P166=D THEN virus=LP | 88.5 | 96 | 52 |
|  | IF P22=F AND P82=S AND P89=T AND P113=R THEN virus=LP | 87.6 | 97 | 52 |
|  | IF P55=E AND P73=S AND P82=S AND P89=T AND P113=R THEN virus=LP | 89.1 | 92 | 50 |
|  | IF P48=S AND P73=S AND P82=S AND P166=D THEN virus=LP | 86 | 100 | 52 |
|  | IF P48=S AND P82=S AND P166=D THEN virus=LP | 85.3 | 102 | 53 |
|  | IF P82=S AND P107=A AND P113=R THEN virus=LP | 85.9 | 99 | 52 |
|  | IF P73=S AND P82=S AND P113=R THEN virus=LP | 85.9 | 99 | 52 |
|  | IF P82=S AND P113=R THEN virus=LP | 85.1 | 101 | 52 |
|  | IF P82=S AND P212=K THEN virus=LP | 81.9 | 105 | 52 |
|  | IF P27=M THEN virus=LP | 83 | 100 | 51 |

Table S8: The strongest rules from the NS2 classifier.

|  | Rule | Accuracy  (%) | Support | Class-Specific-Coverage (%) |
| --- | --- | --- | --- | --- |
| **HP-Rules** | IF P166=A THEN virus=HP | 100 | 167 | 77 |
|  | IF P232=N THEN virus=HP | 100 | 160 | 74 |
|  | IF P224=N THEN virus=HP | 100 | 120 | 56 |
|  | IF P121=T AND P168=I THEN virus=HP | 100 | 119 | 55 |
| **LP-Rules** | IF P49=V AND P60=S THEN virus=LP | 81.8 | 44 | 64 |

Table S9: The strongest rules from the M1 classifier.

|  | Rule | Accuracy (%) | Support | Class-Specific-Coverage (%) | |
| --- | --- | --- | --- | --- | --- |
| **HP-Rules** | IF P166=A THEN virus=HP | 99.4 | 159 | 91 |  |
|  | IF P232=N THEN virus=HP | 100 | 153 | 88 |  |
|  | IF P224=N THEN virus=HP | 99.3 | 144 | 82 |  |
|  | IF P121=T AND P168=I THEN virus=HP | 100 | 127 | 73 |  |
|  | IF P27=K THEN virus=HP | 99.1 | 111 | 63 |  |
|  | IF P168=I THEN virus=HP | 96.2 | 132 | 73 |  |
| **LP-Rules** | IF P166=V AND P232=D THEN virus=LP | 84.1 | 69 | 95 |  |
|  | IF P166=V THEN virus=LP | 79.5 | 73 | 95 |  |
|  | IF P101=R THEN virus=LP | 81.5 | 65 | 87 |  |

Table S10: The strongest rules from the M2 classifier.

|  | Rule | Accuracy  (%) | Support | Class-Specific-Coverage (%) |
| --- | --- | --- | --- | --- |
| **HP-Rule** | IF P14=E THEN virus=HP | 98.3 | 172 | 85 |
| **LP-Rules** | IF P14=G AND P66=E THEN virus=LP | 80.2 | 86 | 96 |
|  | IF P14=G AND P28=I THEN virus=LP | 83.3 | 66 | 76 |
|  | IF P14=G AND P18=K THEN virus=LP | 80.6 | 67 | 75 |
|  | IF P28=I AND P82=S THEN virus=LP | 79.7 | 64 | 71 |
|  | IF P18=K AND P28=I THEN virus=LP | 80.4 | 56 | 63 |

Table S11: The strongest rules from the NP classifier.

|  | Rule | Accuracy  (%) | Support | Class-Specific-Coverage (%) |
| --- | --- | --- | --- | --- |
| **HP-Rules** | IF P377=N AND P482=N THEN virus=HP | 99.6 | 231 | 73 |
|  | IF P34=S AND P377=N THEN virus=HP | 100 | 220 | 70 |
|  | IF P34=S AND P482=N THEN virus=HP | 100 | 217 | 69 |
|  | IF P77=R AND P482=N THEN virus=HP | 99.6 | 223 | 70 |
|  | IF P77=R AND P377=N THEN virus=HP | 99.5 | 222 | 70 |
|  | IF P34=S THEN virus=HP | 98.3 | 234 | 73 |
|  | IF P373=A AND P450=S THEN virus=HP | 100 | 193 | 61 |
|  | IF P373=A AND P377=N THEN virus=HP | 100 | 186 | 59 |
| **LP-Rules** | IF P77=K AND P353=V AND P377=S THEN virus=LP | 82.1 | 112 | 74 |
|  | IF P450=N THEN virus=LP | 82.5 | 97 | 64 |

Table S12: The strongest rules from the PA classifier.

|  | Rule | Accuracy  (%) | Support | Class-Specific-Coverage (%) |
| --- | --- | --- | --- | --- |
| **HP-Rules** | IF P129=T THEN virus=HP | 1 | 251 | 51 |
|  | IF P58=S THEN virus=HP | 1 | 248 | 51 |

Table S13: The strongest rules from the PB1 classifier.

|  | Rule | Accuracy  (%) | Support | Class-Specific-Coverage (%) |
| --- | --- | --- | --- | --- |
| **HP-Rules** | IF P113=I AND P149=I THEN virus=HP | 100 | 342 | 80 |
|  | IF P14=V AND P113=I THEN virus=HP | 100 | 337 | 78 |
|  | IF P149=I THEN virus=HP | 99.1 | 350 | 81 |
|  | IF P113=I AND P386=K THEN virus=HP | 100 | 331 | 77 |
|  | IF P14=V THEN virus=HP | 98.9 | 348 | 80 |
|  | IF P384=L THEN virus=HP | 99.3 | 301 | 70 |
|  | IF P59=T AND P113=I AND P215=K THEN virus=HP | 100 | 227 | 53 |
|  | IF P113=I AND P215=K THEN virus=HP | 99.1 | 229 | 53 |
| **LP-Rule** | IF P14=A AND P113=V AND P154=G AND P384=S THEN virus=LP | 90.4 | 229 | 83 |

Table S14: The strongest rules from the PB2 classifier

|  | Rule | Accuracy  (%) | Support | Class-Specific-Coverage (%) |
| --- | --- | --- | --- | --- |
| **HP-Rules** | IF P64=I THEN virus=HP | 99.1 | 337 | 71 |
|  | IF P339=T THEN virus=HP | 100 | 261 | 56 |
| **LP-Rules** | IF P64=M AND P478=I THEN virus=LP | 97 | 164 | 58 |
|  | IF P478=I THEN virus=LP | 94.2 | 173 | 60 |

Table S15: The strongest rules from the PB1-F2 classifier.

|  | Rule | Accuracy  (%) | Support | Class-Specific-Coverage (%) |
| --- | --- | --- | --- | --- |
| **HP-Rules** | IF P57=Y THEN virus=HP | 97.7 | 130 | 85 |
|  | IF P48=P THEN virus=HP | 97.5 | 120 | 78 |
| **LP-Rules** | IF P48=Q THEN virus=LP | 84.5 | 142 | 92 |
|  | IF P50=D THEN virus=LP | 85.3 | 136 | 89 |

Table S16: The strongest rules from the NA classifier. The positions here represent the positions in the sequences that have a stalk deletion.

|  | Rule | Accuracy  (%) | Support | Class-Specific-Coverage (%) |
| --- | --- | --- | --- | --- |
| **HP-Rules** | IF P346=N THEN virus=HP | 100 | 513 | 89 |
|  | IF P362=G THEN virus=HP | 100 | 418 | 73 |
|  | IF P269=T THEN virus=HP | 97.1 | 452 | 76 |
|  | IF P250=D THEN virus=HP | 100 | 364 | 63 |
|  | IF P44=H THEN virus=HP | 100 | 330 | 57 |
|  | IF P80=H THEN virus=HP | 100 | 296 | 52 |
| **LP-Rules** | IF P376=N THEN virus=LP | 93.5 | 277 | 87 |
|  | IF P38=K THEN virus=LP | 94.3 | 264 | 84 |
|  | IF P173=V THEN virus=LP | 95.7 | 253 | 82 |
|  | IF P70=P THEN virus=LP | 92.2 | 270 | 84 |
|  | IF P55=I THEN virus=LP | 93.5 | 245 | 77 |
|  | IF P243=I THEN virus=LP | 93.4 | 242 | 76 |
|  | IF P236=L THEN virus=LP | 93 | 230 | 72 |
|  | IF P24=M THEN virus=LP | 92.5 | 227 | 71 |
|  | IF P14=S THEN virus=LP | 90.3 | 237 | 72 |
|  | IF P44=E THEN virus=LP | 93 | 215 | 68 |
|  | IF P250=S THEN virus=LP | 91 | 223 | 69 |
|  | IF P168=K THEN virus=LP | 91.5 | 211 | 65 |
|  | IF P414=T THEN virus=LP | 94.1 | 188 | 60 |
|  | IF P56=E THEN virus=LP | 92.3 | 194 | 60 |
|  | IF P46=S THEN virus=LP | 91.3 | 195 | 60 |
